# Supplementary material for: Involvement and possible role of transglutaminases 1 and 2 in mediating fibrotic signalling, collagen cross-linking and cell proliferation in neonatal rat ventricular fibroblasts
Source: PLoS One. 2023 Feb 27;18(2):e0281320. doi: 10.1371/journal.pone.0281320 (PMC9970086; doi:10.1371/journal.pone.0281320)
Supplement: S1 Table — (DOCX) [file pone.0281320.s001.docx]

**Table S1.** Oligonucleotide sequences of custom-designed primers for quantitative PCR.

| **Gene name** | **Abbreviation** | **Species** | **Primer sequence (5´ to 3´)** | |
| --- | --- | --- | --- | --- |
| α-smooth muscle actin | α-SMA | Rat | F: | AGCCAGTCGCCATCAGGAAC |
|  |  |  | R: | CCGGAGCCATTGTCACACAC |
| [β2 microglobulin](http://www.bloodjournal.org/content/95/10/3102.short) | B2m | Rat | F: | CCGTGATCTTTCTGGTGCTT |
|  |  |  | R: | GTGGAACTGAGACACGTAGC |
| B-cell lymphoma-2 | BCL-2 | Rat | F: | GGATCCAGGATAACGGAGGC |
|  |  |  | R: | ATGCACCCAGAGTGATGCAG |
| BCL-2-associated X protein | BAX | Rat | F: | CTCCCCGTGAGGTCTTCTTC |
|  |  |  | R: | TCCAGTGTCCAGCCCATGAT |
| Connective tissue growth factor | CTGF | Rat | F: | CAAGGGTCTCTTCTGCGACT |
|  |  |  | R: | GTACACGGACCCACCGAAG |
| Connexin 43 | CX 43 | Rat | F: | AGGCGTGAGGAAAGTACCAA |
|  |  |  | R: | GCACTCCAGTCACCCATGTC |
| Cyclin D1 | CCND 1 | Rat | F: | AGGGAGATTGTGCCATCCAT |
|  |  |  | R: | AAGACCTCCTCTTCGCACTTC |
| Cyclin E2 | CCNE 2 |  | F: | TCTGCATTCTGACCTGGAACC |
|  |  |  | R: | GGTAATCCCAATGAGTTGAAGCA |
| Matrix metalloproteinase-2 | MMP-2 | Rat | F: | AAGAGGCCTGGTTACCCTGT |
|  |  |  | R: | AAGTAGCACCTGGGAGGGAT |
| Matrix metalloproteinase-9 | MMP-9 | Rat | F: | TCCAGTAGACAATCCTTGCAATGTG |
|  |  |  | R: | CTCCGTGATTCGAGAACTTCCAATA |
| Periostin | Periostin | Rat | F: | CTGCCCCGGCTATATGAGAA |
|  |  |  | R: | TGTTGAGTGGTCGTGGCTC |
| Transforming growth factor-β1 | TGF-β1 | Rat | F: | CCATGACATGAACCGACCCT |
|  |  |  | R: | TGCCGTACACAGCAGTTCTT |

F: Forward; R: Reverse.
