# Supplementary material for: Involvement and possible role of transglutaminases 1 and 2 in mediating fibrotic signalling, collagen cross-linking and cell proliferation in neonatal rat ventricular fibroblasts
Source: PLoS One. 2023 Feb 27;18(2):e0281320. doi: 10.1371/journal.pone.0281320 (PMC9970086; doi:10.1371/journal.pone.0281320)
Supplement: S1 Graphical abstract — (PPTX) [file pone.0281320.s002.pptx]

## Slide 1
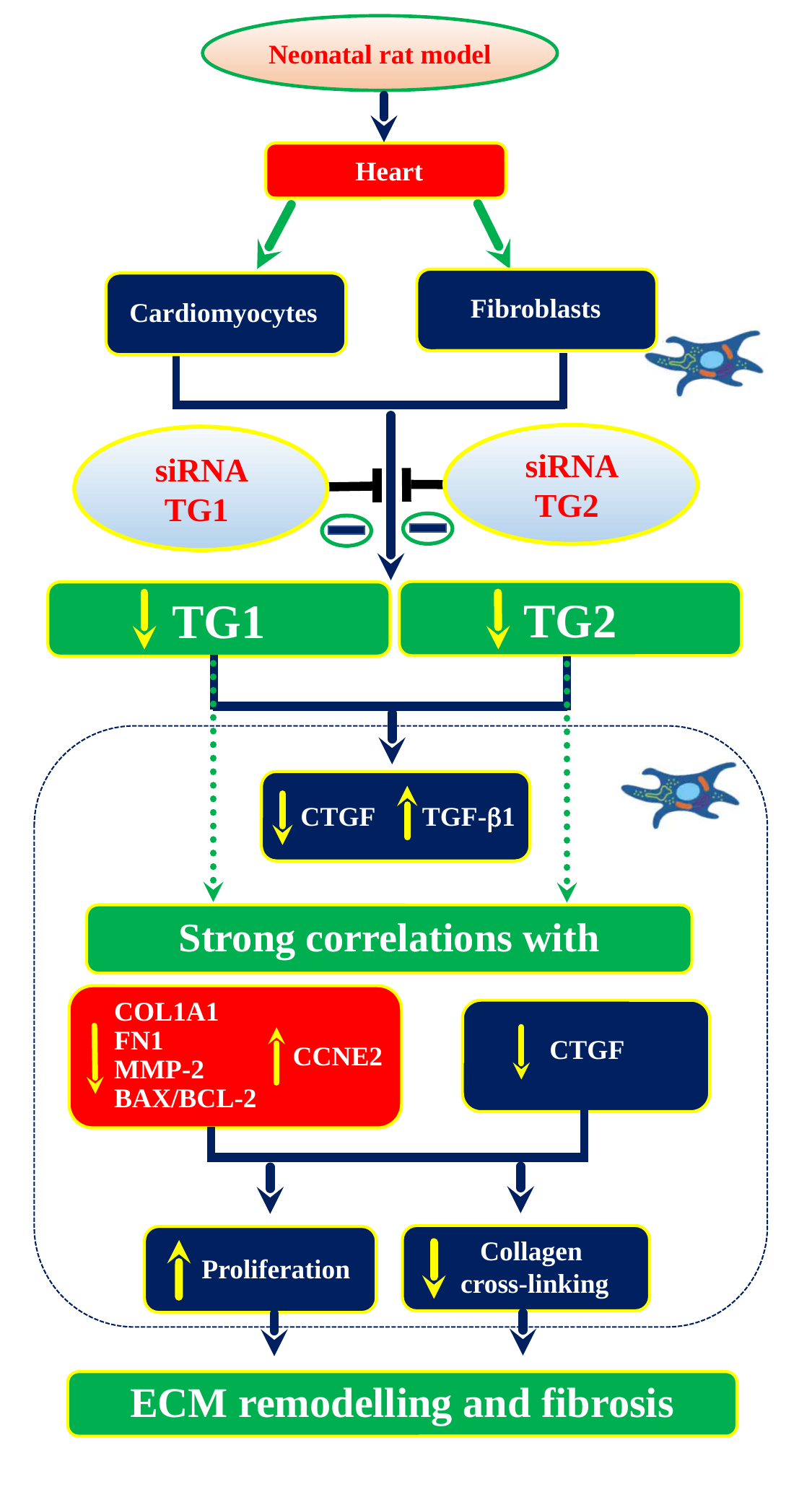

Neonatal rat model
 Heart
Cardiomyocytes
siRNA TG2
siRNA TG1
TG2
TG1
CTGF TGF-b1
Strong correlations with
COL1A1
FN1
MMP-2
BAX/BCL-2
CCNE2
CTGF
Collagen
cross-linking
Proliferation
ECM remodelling and fibrosis
 Fibroblasts
